# Advancing the Science of Education Through Peer Review: Acknowledging Our Reviewers

**DOI:** 10.1212/NE9.0000000000200324

**Published:** 2026-05-13

**Authors:** Roy E. Strowd, Asif Doja

Peer review remains one of the most important and often underrecognized forces advancing science in medical education. It is through this process that ideas are refined, methods are strengthened, and conclusions are challenged and clarified before entering the scholarly record. In a field where our work directly shapes how we teach, learn, and ultimately care for patients, the stakes of rigorous review could not be higher.

At *Neurology® Education*, peer reviewers serve not simply as evaluators but as partners in scholarship. Their thoughtful critiques elevate the quality of submissions, ensuring that the work we publish reflects methodological rigor, conceptual clarity, and meaningful relevance to educators and clinicians. In doing so, reviewers help transform individual manuscripts into contributions that can truly advance the field.

Over the past year, we have been privileged to work with an ever-growing and continuously dedicated community of reviewers that includes clinicians, educators, researchers, and trainees, each bringing unique expertise and perspective. Their efforts ensure that the science of medical education continues to evolve thoughtfully and responsibly. This work is voluntary, often invisible, and always essential. It is the foundation on which trust in academic publishing is built.

Peer review does more than safeguard quality; it is essential to ensuring that scientific discourse remains robust, data-driven, and grounded in evidence. Now more than ever, it plays a critical role in challenging existing assumptions, elevating diverse perspectives, and fostering open, constructive dialogue rooted in data rather than opinion. In medical education, there are often few opportunities for formal peer review of teaching materials, educational outcomes, and new instructional methods. It is precisely these domains where rigorous, thoughtful critique is most needed to ensure that innovation is not only creative, but effective, generalizable, and grounded in evidence. In this way, peer review is not a checkpoint at the end of the scientific process; it is an integral part of teaching discovery itself.

We are deeply grateful to the reviewers who contributed their time and expertise this year. Your work strengthens not only this journal but also the broader community of educators committed to improving neurologic education.

As we look ahead, the landscape of academic publishing continues to evolve, shaped by new technologies, changing dissemination models, and increasing demands for transparency and impact. Through all of this, the role of the peer reviewer remains constant: to uphold standards, to advance science of teaching and training, and to ensure that what we publish meaningfully improves how we teach and how we care for patients.

To our reviewers: thank you. Your contributions are the reason this work matters.

Top Reviewers of 2025

Editorial Board Members:

Elizabeth A. Coon

Zachary N. London

Andrew M. Southerland

Robert Thompson Stone

Stephen M. Sozio

Ad Hoc Reviewers:

Jorge Patino

Steven M. Lazar

Cheran Elangovan

Daniel S. Harrison

Nicolas A. Morris

The list below includes reviewers who returned a review between January 1, 2025, and December 31, 2025. Reviewers with 1 asterisk have reviewed 5 or more articles while 2 asterisks indicate that the reviewer has reviewed 10 or more articles.

Paula Adler

Dara V.F. Albert*

Catherine S.W. Albin

Chaitanya Amrutkar

Xiaoxian An

Corrado I. Angelini

Aileen A. Antonio

Suhas S. Bajgur

Gerald Bean

James L. Bernat

Miya E. Bernson-Leung*

Anand K. Bery

Sonal Bhatia

Tyson Burghardt

Richard B. Carozza

Joseph Carrera

Nikita Chhabra

Kelly Conner*

Elizabeth A. Coon**

Tirth Dave

Chetna Dengri*

Shefali Dujari

Cheran Elangovan*

Andres Fernandez*

Jessica R. Fesler

Anthony L. Fine

Jenna Ford

Katherine A. Fu

Prasanna Kumar Gangishetti

Joseph R. Geraghty

Shivani Ghoshal

Jaleed A. Gilani

Chris Gillette

Raja Godasi

Jessica H.R. Goldstein*

Rachel Gottlieb-Smith*

Patricia Jokl Graese

Scott N. Grossman

Preeta Gupta

Deepak K. Gupta

Daniel S. Harrison*

April Henry

Alissa S. Higinbotham

Chloe E. Hill

Sarah Hodges

Brendan Huang*

Nuri Jacoby

Sleiman El Jamal

Peter H. Jin

Lyell K. Jones

Renzo Figari Jordan

Ralph F. Jozefowicz

Tamara Kaplan

Rahim Abo Kasem

Wesley T. Kerr

Amer Al khalifa

Laura Kirkpatrick

Elizabeth Kouzmitcheva

Michael Shang Kung

Clare McGarvey Lambert

Douglas P. Larsen*

Steven M. Lazar*

Stephane Legriel

Aaron Levy

Grant L. Lin

Jonathan L. Liss

Emma Monti Loebel*

Zachary N. London**

Max R. Lowden

Marie Charmaine Sy Lukban*

Ariel M. Lyons-Warren

Vincenzo Marcelli

Nina Massad

Tomoyasu Matsubara

Rebecca K. Miller-Kuhlmann

Miguel Silva Miranda

Murli Mishra

Jeremy J. Moeller*

Teshamae S. Monteith

Daniel Moreno-Zambrano

Nicholas A. Morris*

Vandana Nagpal

Divya Nayar

Jordan H. Newman

Kshama Ojha

Setareh Salehi Omran

Frédérique Ouellet

Gustavo A. Patino

Jorge Patino**

Pritikanta Paul

Michael A. Perez

Matthieu Paul Perrenoud

Stephen Joseph Powell

Hira Rafi

Subha Ramani

Jeffrey B. Ratliff

Margie A. Ream

Rachel Marie E. Salas

Stefano Sandrone

Veronica E. Santini

Deanna R. Saylor

Elke S. Schipani*

Suma Shah

Anita V. Shelgikar

Madhu Soni

Marinos G. Sotiropoulos

Andrew M. Southerland*

Stephen M. Sozio*

Robert Thompson Stone*

Isabella Strozzi

Harry W. Sutherland*

Monica Taing

Christopher Tarolli

Erick Tarula

Zahari N. Tchopev

Liu Lin Thio

Elizabeth T. Troy

Darren Tse

Simone Veronese

Ericka Shin Wong

Kathryn Xixis

Edward H. Yu

Aaron S. Zelikovich

Jiping Zhou

